# Enhanced Light–Matter Interaction in Metallic Nanoparticles: A Generic Strategy of Smart Void Filling

**DOI:** 10.1021/acs.nanolett.4c00810

**Published:** 2024-04-05

**Authors:** Changxu Liu, Tong Wu, Philippe Lalanne, Stefan A. Maier

**Affiliations:** † Centre for Metamaterial Research & Innovation, Department of Engineering, University of Exeter, Exeter EX4 4QF, United Kingdom; ‡ LP2N, Institut d’Optique Graduate School, CNRS, Université de Bordeaux, Talence 33400, France; ¶ School of Physics and Astronomy, Monash University, Clayton, Victoria 3800, Australia; § Blackett Laboratory, Imperial College London, London SW7 2BZ, United Kingdom

**Keywords:** plasmonics, nanoparticles, transition metals, light−matter interaction

## Abstract

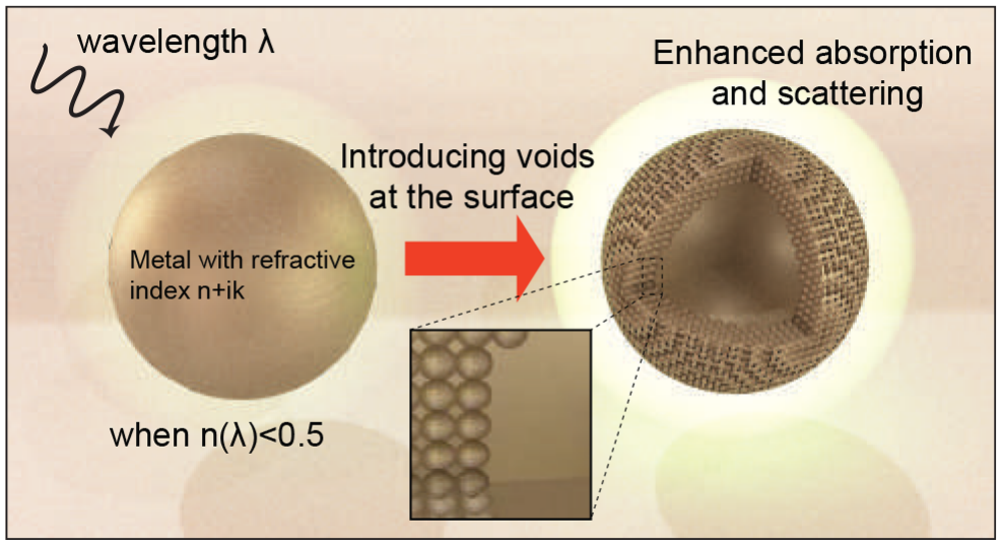

The intrinsic properties of materials play a substantial role in light–matter interactions, impacting both bulk metals and nanostructures. While plasmonic nanostructures exhibit strong interactions with photons via plasmon resonances, achieving efficient light absorption/scattering in other transition metals remains a challenge, impeding various applications related to optoelectronics, chemistry, and energy harvesting. Here, we propose a universal strategy to enhance light–matter interaction, through introducing voids onto the surface of metallic nanoparticles. This strategy spans nine metals including those traditionally considered optically inactive. The absorption cross section of void-filled nanoparticles surpasses the value of plasmonic (Ag/Au) counterparts with tunable resonance peaks across a broad spectral range. Notably, this enhancement is achieved under arbitrary polarizations and varied particle sizes and in the presence of geometric disorder, highlighting the universal adaptability. Our strategy holds promise for inspiring emerging devices in photocatalysis, bioimaging, optical sensing, and beyond, particularly when metals other than gold or silver are preferred.

Structured nanoparticles composed of various metals are pivotal in the fields of photonics, chemistry, materials science, and biomedicine. Among their diverse applications, interaction among nanoparticles and photons across different spectral ranges has given rise to numerous possibilities, spanning from chemical/bio sensing, imaging to photocatalysis and photothermal therapy.^1−5^ To achieve optimal performance, a standard approach is to enhance the light–matter interaction within or around metallic nanostructures, aiming to maximize the absorption/scattering cross section for individual nanoparticles.

Within the library of transition metals, metals with a strong plasmonic effect,^6−8^ such as silver (Ag) and gold (Au), emerge as exemplary candidates for achieving a strong interaction with photons through the excitation of localized surface plasmon resonances (LSPR).^9^ Various designs have been developed to boost scattering and/or absorption at the nanoscale, including nanorods,^10,11^ nanodisks,^12^ nanocages,^13^ core–shell structures,^14−17^ and coupled structures with narrow gaps,^18,19^ sharp tips, or connections.^18−22^ However, trade-offs among the desirable characteristics may be needed, including large values of cross section, polarization insensitivity, broadband response with adjustable resonance peak, and large surface to volume ratio.

Moreover, most of the strategies developed have predominately focused on Ag/Au based nanoparticles, leaving the improvement of light–matter interactions in nonplasmonic metals as an elusive question. Nanostructures composed of various nonplasmonic metals, with distinct chemical properties, excel in various catalysis and nanomedicine-related applications.^23−26^ However, this superior performance encounters significant hindrances when visible or infrared photons are employed as stimuli owing to reduced light absorption and/or scattering. To address the challenge of enhancing the light–matter interaction of nonplasmonic metals, some compromise methods have been developed, involving hybrid metallic configurations, where plasmonic nanoparticles are introduced in the vicinity of optically inactive nanostructures^27−38^ but only offering moderate enhancements.

In this Letter, we present a universal method for enhancing light–matter interactions in nanoparticles composed of various transition metals, including those traditionally considered optically inactive. By introducing voids into the outer part of the nanoparticle (as shown in Figure 1), we achieve significant enhancement in absorption of single nanoparticles composed of nine types of metals, including silver (Ag), aluminum (Al), gold (Au), cobalt (Co), copper (Cu), nickel (Ni), palladium (Pd), platinum (Pt), and rhodium (Rh). We observe a remarkable increase in absorption cross section for Au (Ag) within a broad spectrum from visible to infrared. More interestingly, the effect of absorption enhancement extends to the other seven metals, all surpassing the “best” plasmonic (Ag/Au) spherical counterparts. The universal adaptability and versatility of our method is highlighted by its effectiveness under arbitrary polarization, different particle sizes, and even in the presence of geometric disorder (shape deformations of the nanoparticle), through Mie theory and FDTD simulations. We anticipate that our method will catalyze further experimental investigations, opening avenues for the use of meta-particles (shape-engineered particles with unprecedented properties) with enhanced light–matter interaction. Its potential extends to improving the performance of devices in photocatalysis, bioimaging, optical sensing, and beyond, particularly in scenarios where metals other than gold or silver are preferred.

**Figure 1:**
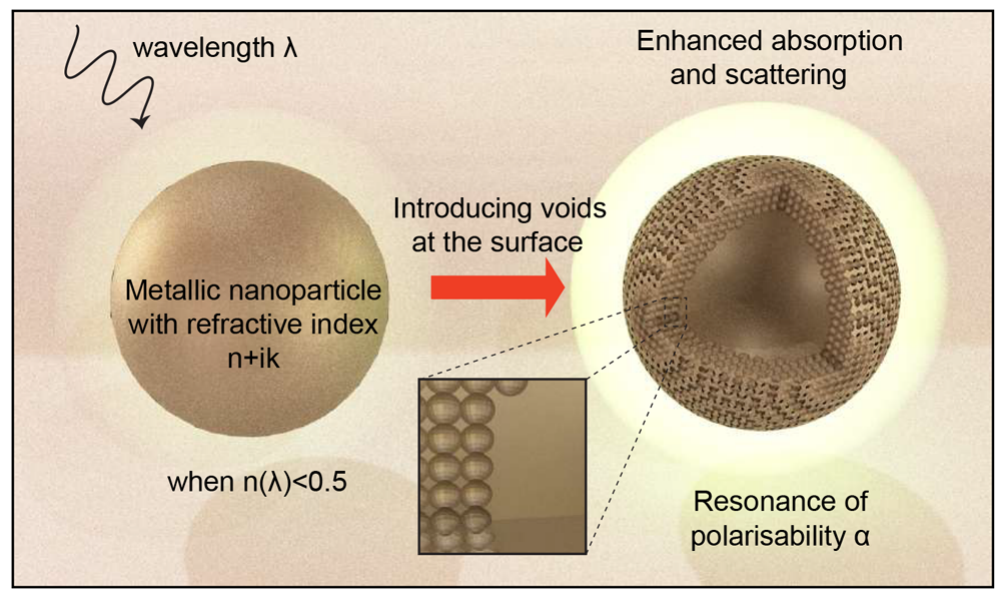
Conceptual schematic of voiding filling enhanced light–matter interaction of a metallic nanoparticle. λ is the wavelength, and *n* is the real part refractive index of the metal.

We initiated our investigation using a spherical core–shell structure as the toy model, illustrated in the inset of Figure 2a. The core consists of a transition metal with relative permittivity ε_*c*_, while the shell is a material with arbitrary permittivity ε_*s*_ = (*n* + *i k*)^2^, with *n* the real part of the refractive index and *k* the imaginary part. The background is chosen as air with permittivity ε_bg_ = 1. For simplicity but not losing generality, we choose a gold nanosphere as the core, with the radius *R*_*i*_ = 50 nm. The thickness of the shell is chosen as 15 nm, i.e., the radius of the whole sphere *R*_*o*_ = 65 nm.

**Figure 2:**
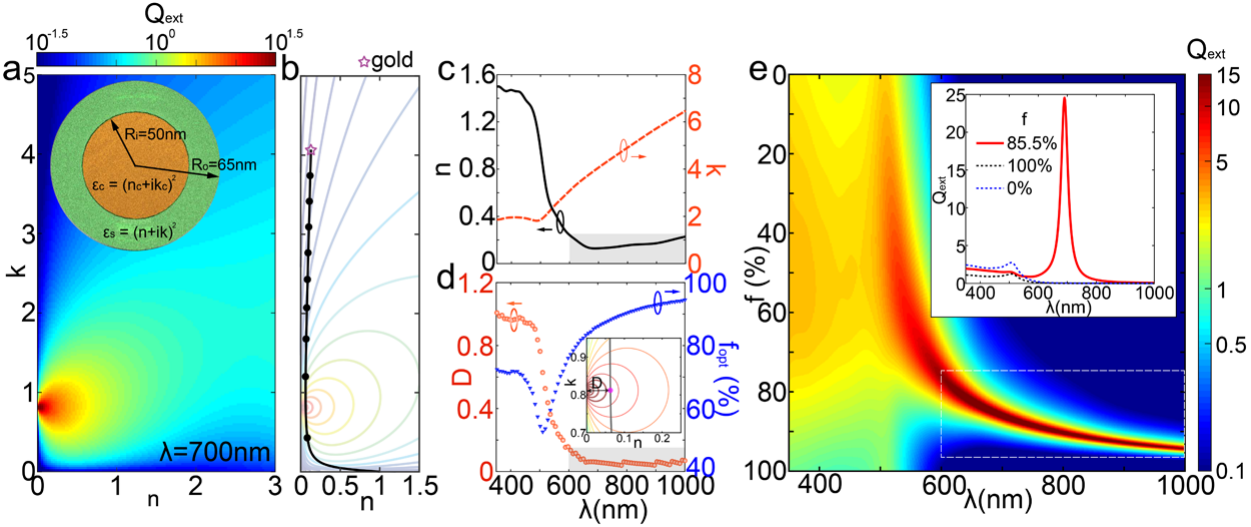
Demonstration of void-filling-enhanced light–matter interaction based Mie theory. (a) Calculated values of *Q*_ext_ in the complex refractive index plane *ñ* = *n* + *ik*. Inset shows the configuration of the core–shell structure, with *R*_*o*_ = 65 nm and *R*_*i*_ = 50 nm. (b) The trajectory (black solid line) of *ñ*_eff_ with the increment of *f*, the filling factor of the air. The starting point is denoted by the magenta star with a refractive index of gold and the end point is *ñ* = 1. The background displays the contour of *Q*_ext_. The black solid circles represent the values of *f* from 0.1 to 0.9 with a step of 0.1 from top to bottom. (c) Complex refractive index of gold as a function of wavelength (λ). (d) The minimum distance (*D*) between the resonant point and *ñ*_eff_ trajectory at different wavelengths. The inset demonstrates the definition of *D* (zoomed in from b). The filling factor *f*_*opt*_ for the minimum value of *D* is also included. (e) *Q*_ext_ spectra with varying values of *f*. Inset illustrates the case with the maximum value of *Q*_ext_. Spectra with *f* = 0 (65 nm Au nanosphere) and *f* = 100% (50 nm Au nanosphere) are presented as references. The spectral regions with small values of *n* (shaded area in c), small values of *D* (shaded area in d) and large values of *Q*_ext_ (area inside white dashed box) overlap.

Under electrostatics approximation, the extinction cross section (σ_*ext*_) can be calculated using Mie theory:^39−42^
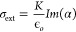
1with *K* the wavevector, ϵ_*o*_ the vacuum permittivity, and α the polarizability. The polarizability is a function of the material properties and geometry:^41,42^
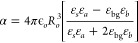
2with ε_*a*_ = ε_*c*_(3–2 *P*) + 2 ε_*s*_*P*, ε_*b*_ = ε_*c*_*P* + ε_*s*_(3 – *P*), and *P* = 1 – (*R*_*i*_/*R*_*o*_)^3^.

Correspondingly, the extinction of the core–shell structure can be calculated on the parametric plane of the complex refractive index of the shell, as illustrated in Figure 2a. The wavelength of the impinging light (λ) is set at 700 nm. The core is gold with permittivity ε_*c*_ = −16.55 + 1.05*i*. To facilitate comparison with nanoparticles of varying sizes, we employ the extinction efficiency factor *Q*_ext_ = σ_*ext*_/π*R*_*o*_^2^. A notable enhancement is observed around *ñ*_*r*_ = (0, 0.8), corresponding to a sharp resonance point with large value. A shell with a refractive index around this reddish region with *Q*_ext_ > 10 can drastically improve the light–matter interaction. However, it is impractical to achieve the desirable value using natural materials with ease of fabrication.

Instead, we present a feasible approach to realize values close to the resonance without the need of additional materials. Initially, we opt for the shell to be made of the same material as the core (Au). Uniformly distributed voids are then introduced to the shell, as shown in Figure 1. By adjusting the fill factor (*f*), the effective refractive index (*ñ*_eff_) can be tuned from that of pure gold to air, as shown in Figure 2b. The black solid line depicts the evolution of *ñ*_eff_ as a function of filling factor, based on Maxwell–Garnett approximation:^43−45^
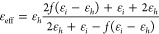
3where the effective permittivity ε_eff_ = *ñ*_eff_^2^, ε_*h*_ is the permittivity of the host medium, and ε_*i*_ is the permittivity of the inclusion. Here, we assume the size of the voids is orders of magnitude smaller than the wavelengths in free space, so the effective permittivity is a function of filling factor and permittivity of the host and inclusion only, independent of the void size.^45^ Interestingly, the curvature of line facilitates the trajectory of *ñ*_eff_ approaching the resonance point. We term this phenomenon “smart void filling”, as the strong extinction of the nanoparticle can be achieved by simply tuning the value of *f*, or equivalently, graduating, introducing voids into the outer part of the nanostructure.

Similar investigations are conducted for impinging light at different wavelengths, and the results are presented in Supplementary Section 1. Both the real and imaginary parts of the refractive index of gold are shown in Figure 2c. The values of *n* and *k* at each wavelength determine the starting point of the *ñ*_eff_ trajectory, indicated by the magenta star in Figure 2b. When the value of *n* is small (the shaded region in Figure 2c), the *ñ*_eff_ trajectory can pass through the region in the vicinity of resonance. On the other hand, the value of *k* plays a minute role in determining whether the trajectory goes close to the resonance. Supplementary Section 1 provides further details, illustrating trajectories for different wavelengths. Figure 2d provides quantitative information about the effect. *D* is defined as the minimum distance between the resonant point *ñ*_*r*_ and the *ñ*_eff_ trajectory, as shown in the inset of Figure 2d. The optimal filling factor (*f*_opt_) is the value at which this minimum distance occurs. A spectral overlap (600 to 1000 nm) is observed where both *n* and *D* are small (shaded regions in Figure 2c,d), irrespective to the variation of *k* going from 3.1 to 6.5. However, as the value of *n* increases below 600 nm, the trajectory cannot approach the desired region, resulting in an increment of *D*.

Figure 2e summarizes the *Q*_ext_ spectra under various filling factors. Void filling leads to a significant enhancement of extinction when *D* is small, contrasting sharply with nanospheres of 50 nm (*f* = 100%) and 65 nm (*f* = 0). The extinction improvement is particularly pronounced between 600 and 1000 nm, as indicated by the region within the white dashed box. This also aligns with the spectrum featuring a small *n* (*D*). The inset of Figure 2e highlights a scenario with a maximum enhancement. At 690 nm, the void filling effect bestows the structure with an extinction efficiency of 24.6, representing a nearly 3 order improvement compared to a nanosphere of the same size (0.04). Simultaneously, there is a 910% enhancement in the peak value compared to the spherical counterpart (2.7 at 506 nm). Interestingly, the void filling also provides the flexibility to tune the position of maximum extinction in a broad range. Increasing the value of *f* from 79% to 94% introduces a red shift from 617 to 960 nm while maintaining the peak value above 15.

To illustrate that the effect of smart filling is not limited to some specific geometries, we conduct additional investigations with different values of *P* = 1 – (*R*_*i*_/*R*_*o*_)^3^. Also, the enhancement of the light interaction is confirmed in a different background environment with *n*_bg_ = 1.33. More details can be found in Supplementary Section 2.

Next, we applied the same strategy of void filling to other transition metals beyond gold. The results are summarized in Figure.3, which illustrates the spectra of *Q*_ext_ for eight different metals: Ag, Al, Co, Cu, Ni, Pd, Pt, and Rh. The inset in each panel shows the case at a specific filling factor with maximum *Q*_ext_. Despite the variations in the maximum values of *Q*_ext_, a universal enhancement in the light–matter interaction is observed for all metals. For Cu and Ni, which show the comparatively moderate improvement, there is an 363% and 379% increase in the peak value of *Q*_ext_, as depicted in the insets of Figure 3d,e, respectively. Similarly, the spectral regions with significant enhancement overlap with the wavelengths exhibiting small values of *n*, demonstrating the same effect demonstrated for Au. The complex refractive indices for the metals used are plotted in Supplementary Section 3. Within this region, the spectral position of the extinction peak can be extended to longer wavelengths (reddish and yellowish tails) and controlled feasibly through the variation of *f*. In sharp contrast, extinction peaks are limited to wavelengths below 400 nm for spherical particles with radius between 50 nm (*f* = 100%, blue dashed lines in the insets) and 65 nm (*f* = 0, black dashed lines in the insets). Less enhancement is observed for Al and Rh in the visible and near-infrared regions, due to the relative large value of *n*, as illustrated in Figure S4. This prevents a small value of *D* from approaching the optimal point in the (*n*, *k*) space.

**Figure 3:**
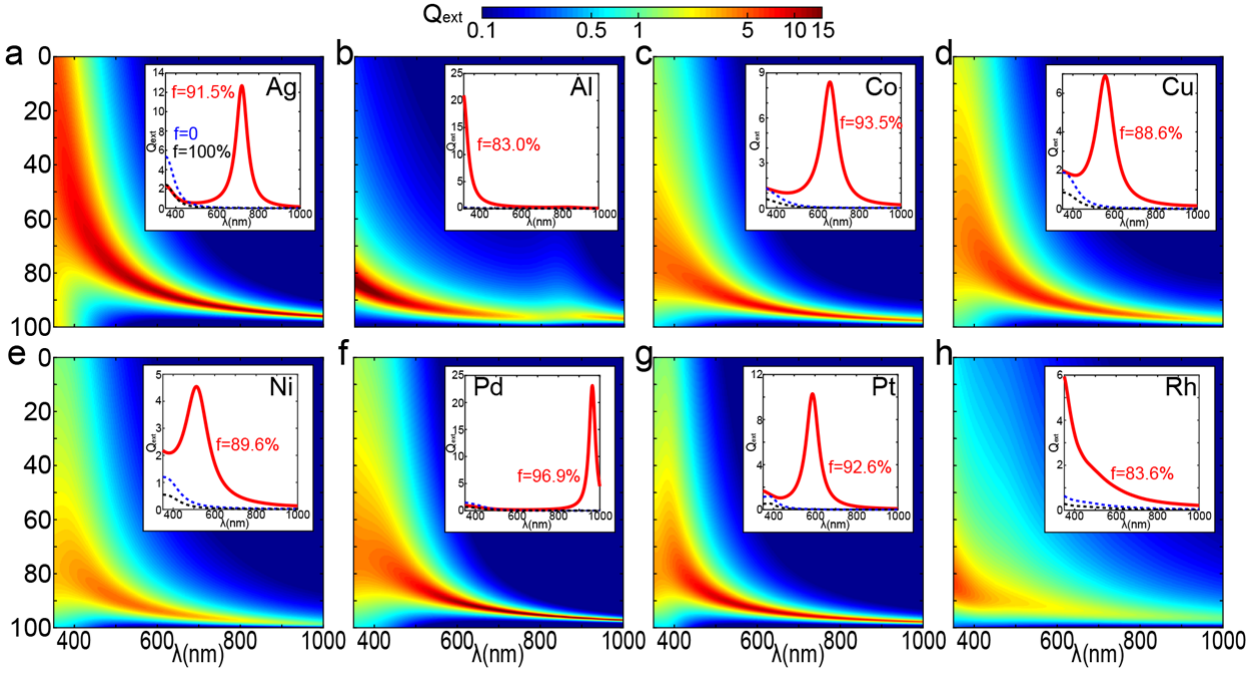
Void filling induces extinction enhancement for transition metals beyond Au. *Q*_ext_ spectra at varying *f* are demonstrated for (a) Ag; (b) Al; (c) Co; (d) Cu; (e) Ni; (f) Pd; (g) Pt; and (h) Pt. The inset demonstrates the spectra with the largest value of *Q*_ext_. The corresponding values of *f* are also labeled.

Inspired by the toy model, we propose realistic structures and conduct comprehensive full-wave simulations, investigating the light–matter interactions without approximation from Mie theory. Initially, we designed a nanostructure comprising a 50 nm spherical core with uniformly distributed spikes on its surface, as shown in Figure 4a. The symmetry guarantees a polarization-independent optical response. As the number of the spikes (*N*_spk_) increases, the filling factor of the voids *f* discretely goes from 100% to 0. In the limit of an infinite number of spikes, the structure evolves into a sphere with a radius of 65 nm. We term this structure “nanourchin” due to its resemblance to a sea urchin, particularly with a moderate number of spikes.

**Figure 4:**
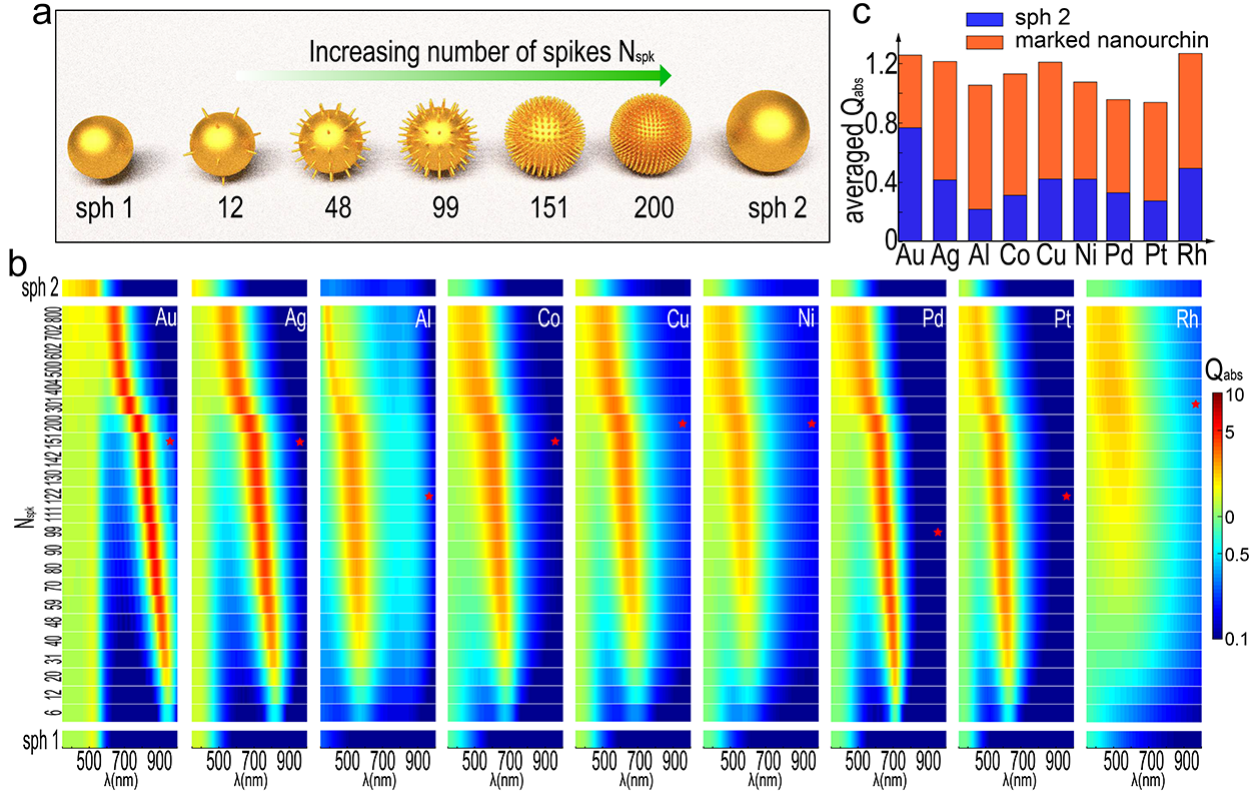
Design for the realization of void filling and enhanced light absorption in nine metals. (a) Schematic of proposed nanourchins composed of a spherical core with attached spikes. The filling factor can be tuned by varying the number of spikes (*N*_spk_). Sph1 (sph2) represents nanourchins with zero (infinitive) numbers of spikes. (b) The *Q*_abs_ spectra of nanourchins with different *N*_spk_. Nine metals are included, with the type listed at the top right of each panel. The red star marks the nanourchin with maximum value of *Q*_abs_. (c) Averaged values of *Q*_abs_ within a broad spectrum between 300 and 900 nm.

Here, we focus on the absorption efficiency *Q*_abs_, which directly determines the energy conversion efficiency in photovoltaic, photocatalysis, and photothermal conversion. The spectra of extinction efficiency can be found in Supplementary Section 4. In Figure 4b, we present simulations showing the *Q*_abs_ values for different metals with varying numbers of spikes. The bottom and top panels serve as references for the *Q̅*_abs_ values of nanospheres. A universal enhancement of absorption is observed for all nine materials, including both plasmonic metals and catalytic ones with week optical responsive. Meanwhile, the tunability of the absorption peak is confirmed, where the reddish regions experience a redshift as the decrease of *N*_spk_. This is particularly significant for solar energy harvesting, as a considerable portion of energy resides in the 600 to 1000 nm region. Nanourchins with proper filling factors can efficiently covers this regime, which is not feasible with classical structures of the same size. Similar enhancement of scattering is also observed, with details shown in Supplementary Section 4.

Quantitative comparison in broadband response between nanourchins and nanospheres of the same size is presented in Figure 4c. Here, the averaged value (*Q*_abs_) between 350 and 1000 nm is presented. Prominent enhancement is demonstrated for all metals. Intriguingly, all nanourchins surpass the value of the best plasmonic nanosphere (Au), highlighting that void filling can endow stronger interaction for all metals beyond a plasmonic standard.

To prove the universal adaptability, we investigate the cases with different geometries, in a different host material and existence of the disorder, as summarized in Figure 5. Here, we choose gold as the materiel of all nanourchins. The scalability predicted by our Mie model (in eq 2 where only the ratio *R*_*i*_/*R*_*o*_ contributes) is investigated, as shown in Figure 5a. The *Q*_abs_ spectra of nanourchins with two different sizes are provided, one with doubled size (130 nm, right panel) and the other with half size (32.5 nm, left panel) of the original one (65m) shown in Figure 3b. The void-filling-induced absorption enhancement under different scales is clarified, while a red (blue) shift of the resonance is observed for the structure of doubled (halved) size. Figure 5b illustrates the case when the nanourchin is embedded in a background with refractive index of 1.33. A prominent redshift is demonstrated to absorb more light in the near IR.

**Figure 5:**
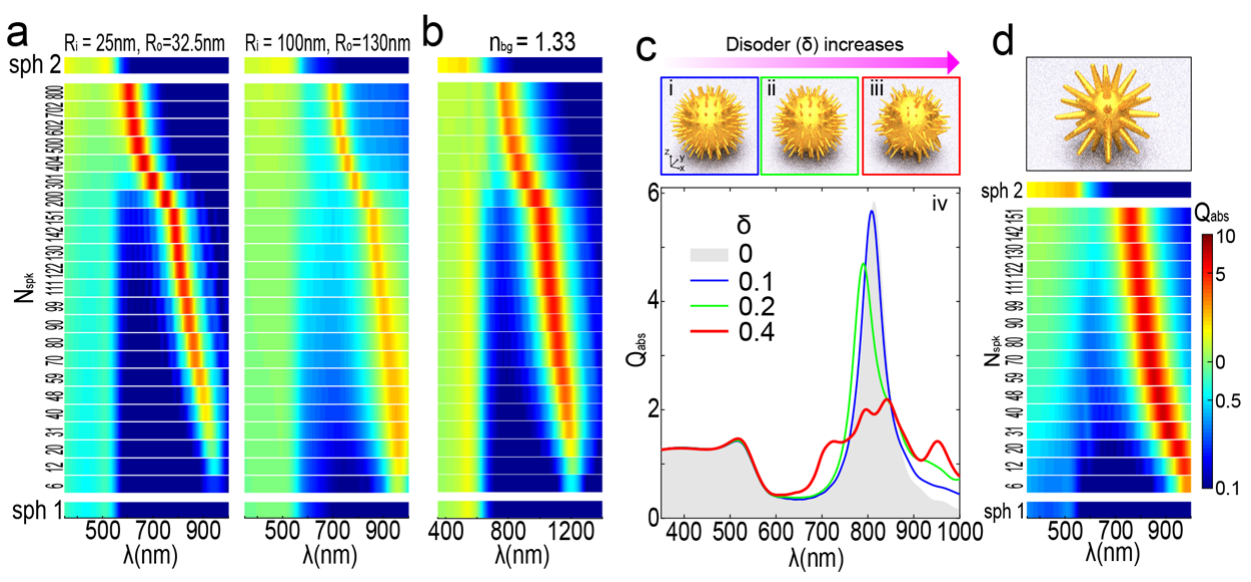
Universal adaptability of smart void filling. (a) *Q*_abs_ spectra of Au nanourchins with two different sizes: 32.5 nm (left panel) and 130 nm (right panel) (b) *Q*_abs_ spectra of Au nanourchins inside a host material with *n*_bg_ = 1.33. (c) *Q*_abs_ spectra of Au nanourchins when disorder is introduced. Panels (i)–(iii) demonstrate the geometry of nanourchin with increasing levels of disorder (δ = 0.1, 0.2, and 0.4). (d) *Q*_abs_ spectra of Au nanourchins with smaller core and longer spikes. The top panel is a schematic of the geometry, and the bottom panel shows the spectra.

In real-world scenarios, structural imperfections are inherent at subwavelength scale. Accordingly, we investigate the impact of geometric disorder for our proposed nanourchins. We systematically introduce fluctuations into the size and orientation of the spikes, illustrated in panels (i)–(iii) of Figure 5c, where δ represents the level of disorder. Disorder is introduced in both size (along longitudinal direction and within transverse plane) and orientation of the spikes of the nanourchin, with more details shown in Supplementary Section 5. The corresponding *Q*_abs_ is depicted in Figure 5c(iv), with the shaded background representing the case without disorder. A linear polarized light is used with the electric field aligned along the *x*-axis shown in Figure 5c (i). The introduction of disorder leads to a reduction in peak value from 5.85 to 2.19. However, it promotes absorption across a broader spectral range.^46^ The relative change of averaged value, *Q̅*_abs_(δ)/*Q̅*_abs_(δ = 0), is 102.5% for (i), 105.5% for (ii), and 98.6% for (iii), respectively. Another set of simulations, employing a different set of random variables, is conducted to illustrate that the observed effect is not due to a specific geometry, with more details shown in Supplementary Section 6. To provide more info related to practical applications, we further investigate the polarization dependence of the nanoparticles and examine the nanoparticles with dielectric coatings, with details provided in Supplementary Sections 7 and 8, respectively.

Finally, we present an alternative realization for blackberry-like nanoparticles, composed of a core nanoparticle with secondary nanoparticles affixed, demonstrating similar optical response (Supplementary Section 9). A comparison between nanourchin and nanoblackberry is presented in Supplementary Section 10, illustrating the influence of specific morphology on the optical response.

Utilizing a combination of analytical modeling and full wave simulations, our study introduces and demonstrates the impactful effect of void filling on enhancing light–matter interaction for metallic nanoparticles. The versatility of the approach is elucidated across nine different transition metals, various sizes, and even with disorder in the geometry. While it has been previously reported that nonspherical nanoparticles composed of various transitional metals exhibit improved light–matter interactions compared to Au nanospheres,^47^ we provide a new perspective through modeling within the (*n*, *k*) space, which encapsulates complex geometric features into a single refractive index value. This approach clarifies the design methodology for achieving optimal performance and allows for the manipulation of resonance within the desired spectra. Furthermore, our findings suggest that the material library for strong light–matter interactions may be expanded beyond metals to include alloys and compounds, particularly in scenarios where small values of *n* are present.

The proposed design presents a potential complement to the existing nanostructure library for light–matter interactions, encompassing nanorods, nanodisks, nanocages, core–shell structures, and coupled structures with narrow gap, sharp tips, or connections. While nanostructures with gaps, sharp tip/connection, and nanorods exhibit a strong dependence on the polarization of incident light, core–shell structures and disks struggle to adjust resonance without altering their physical cross section. Singular plasmonic structures^48,49^ provide a strong response within a broadband region but are sensitive to polarization, whereas random plasmonic metasurfaces^50−52^ offer a broadband response but lack tunability of the resonance. The strategy of void filling can lead to strong light–matter interactions with feasible resonance tunability. Interestingly, a redshift in absorption peak can even be achieved by reducing the size of the nanoparticle, provided the filling factor is increased from a small value (as shown in Figure 5a,b). Combined with polarization insensitivity, the proposed nanoparticle may find utility in strong plasmon–exciton interaction,^53,54^ where voids can also induce the strong near-field enhancement. Filling-factor-engineered nanoparticles could serve as a platform for achieving both interlayer and intralayer plasmon-exciton strong couplings under unpolarized illumination.

This strategy holds promise for applications in which a strong interaction between photons and nanoparticles is crucial. Void filling not only enlarges the absorption cross section of the plasmonic (Au/Ag) nanoparticles but also extends absorption peaks into the infrared spectrum, potentially overcoming limitations in certain applications. For example, in hot-electron-driven photocatalysis under solar radiation, achieving high and polarization-independent absorption with a high rate of hot electron generation is highly desirable. While traditional size upscaling can cover the red and near-infrared spectrum, it often diminishes the efficiency of energetic carrier generation.^55^ In contrast, void filling introduces a redshift in the absorption peak without enlarging the particle size, providing an efficient approach to harnessing solar energy at longer wavelengths.

More importantly, void filling lights up nanoparticles composed of various transition metals, making them more optically active than Au/Ag nanospheres of the same size. Correspondingly, Pt/Pd/Cu-based nanoparticles can be transformed to efficient absorbers, facilitating the efficient use of hot carriers for photocatalysis. Meanwhile, Au/Ag based nanoparticles may be substituted with earth-abundant metals such as Cu, Ni or Al without sacrificing performance. Also, the strategy can be extended to nanoparticles made of metallic alloys, when elements of the alloy share a small value of *n* in the spectrum (such as PtCo alloy).^56^ Last but not least, the material consumption for absorption-related applications may be significantly reduced, with more details shown in Supplementary Section 11.

Our work may stimulate experimental efforts in realizations for the proposed designs with desired filling factors. Self-assembly based on wet-chemistry may be employed to create nanourchin (Figure 4a)^57−59^ and nanoblackberry (Figure S8a)^60,61^ structures. Cluster beam deposition^62,63^ offers the possibility to overcome the material limitations from chemical synthesis, enabling the fabrication of secondary spheres of the nanoblackberry with different transition metals.^64^ Beyond the proposed shapes, porous shells could be fulfilled through the process of dealloying of outer part of alloy nanoparticles.^65,66^

In this study, we focus on the interaction between light and an individual nanoparticle. However, light–matter interaction may be further enhanced in systems with multiple nanoparticles, through engineering optical environments and interparticle coupling. Combining techniques such as dielectric environment encapsulation,^67^ disordered scattering/coupling^63^ and curvature-induced field enhancement,^68^ the smart filling may inspire diverse nanophotonic devices for photocatalysis, energy harvesting, sensing and beyond.

The model based on the Mie theory treats the shell as an effective medium with a refractive index. However, the shape and distribution of voids play a significant role in determining the optical response, as demonstrated in Figure 5. Therefore, while the Mie model provides initial guidance, further investigation, including numerical simulations, may be necessary for practical design when disorder is inevitable. Disorder in the system can broaden the bandwidth of the optical response,^46,51,64^ offering advantages for specific applications such as photovoltaics and photocatalysis. The modeling and simulations does not include the influence of nanoscale electromagnetism,^69,70^ prompting further investigation of nonlocal effects and electron tunneling for the proposed nanoparticles.

## Supporting Information Available

Trajectory of void filling under different wavelengths for Au nanosphere; universal adaptability of smarting void filling (calculation based on Mie theory); refractive indices for the transition metals; extinction efficiency and scattering efficiency for nanourchins composed of different metals; details of the simluations; impact of disorder on the effect of void filling (another set of random variables); impact of polarization of incident light; impact of dielectric coating; nanoblackberry, alternative design for a realizable naoprticle with void filling; a comparison between nanoblackberry and nanourchin; simple estimation for the reduction of material usage in absorption-related applications (PDF)

## References

[ref1] Sau T. K.; Rogach A. L.; Jäckel F.; Klar T. A.; Feldmann J. Properties and applications of colloidal nonspherical noble metal nanoparticles. Adv. Mater. 2010, 22, 1805–1825. 10.1002/adma.200902557. 20512954

[ref2] Jiang Z.; Le N. D.; Gupta A.; Rotello V. M. Cell surface-based sensing with metallic nanoparticles. Chem. Soc. Rev. 2015, 44, 4264–4274. 10.1039/C4CS00387J. 25853985 PMC4478158

[ref3] Liu L.; Zhang X.; Yang L.; Ren L.; Wang D.; Ye J. Metal nanoparticles induced photocatalysis. National Science Review 2017, 4, 761–780. 10.1093/nsr/nwx019.

[ref4] Gao C.; Lyu F.; Yin Y. Encapsulated metal nanoparticles for catalysis. Chem. Rev. 2021, 121, 834–881. 10.1021/acs.chemrev.0c00237. 32585087

[ref5] Luo X.; Liu J. Ultrasmall luminescent metal nanoparticles: surface engineering strategies for biological targeting and imaging. Adv. Sci. 2022, 9, 2103971 10.1002/advs.202103971. PMC8787435 34796699

[ref6] de Aberasturi D. J.; Serrano-Montes A. B.; Liz-Marzán L. M. Modern applications of plasmonic nanoparticles: from energy to health. Advanced Optical Materials 2015, 3, 602–617. 10.1002/adom.201500053.

[ref7] Wang L.; Hasanzadeh Kafshgari M.; Meunier M. Optical properties and applications of plasmonic-metal nanoparticles. Adv. Funct. Mater. 2020, 30, 2005400 10.1002/adfm.202005400.

[ref8] Montes-García V.; Squillaci M. A.; Diez-Castellnou M.; Ong Q. K.; Stellacci F.; Samori P. Chemical sensing with Au and Ag nanoparticles. Chem. Soc. Rev. 2021, 50, 1269–1304. 10.1039/D0CS01112F. 33290474

[ref9] Maier S. A. Plasmonics: Fundamentals and Applications; Springer, 2007; Vol. 1.

[ref10] Chen H.; Shao L.; Li Q.; Wang J. Gold nanorods and their plasmonic properties. Chem. Soc. Rev. 2013, 42, 2679–2724. 10.1039/C2CS35367A. 23128995

[ref11] Zheng J.; Cheng X.; Zhang H.; Bai X.; Ai R.; Shao L.; Wang J. Gold nanorods: the most versatile plasmonic nanoparticles. Chem. Rev. 2021, 121, 13342–13453. 10.1021/acs.chemrev.1c00422. 34569789

[ref12] Wan W.; Zheng W.; Chen Y.; Liu Z. From Fano-like interference to superscattering with a single metallic nanodisk. Nanoscale 2014, 6, 9093–9102. 10.1039/C4NR02107J. 24975582

[ref13] Skrabalak S. E.; Au L.; Li X.; Xia Y. Facile synthesis of Ag nanocubes and Au nanocages. Nat. Protoc. 2007, 2, 2182–2190. 10.1038/nprot.2007.326. 17853874

[ref14] Ruan Z.; Fan S. Design of subwavelength superscattering nanospheres. Appl. Phys. Lett. 2011, 98, 043101 10.1063/1.3536475.

[ref15] Monticone F.; Argyropoulos C.; Alù A. Multilayered plasmonic covers for comblike scattering response and optical tagging. Phys. Rev. Lett. 2013, 110, 113901 10.1103/PhysRevLett.110.113901. 25166536

[ref16] Antosiewicz T. J.; Apell S. P.; Shegai T. Plasmon–exciton interactions in a core–shell geometry: from enhanced absorption to strong coupling. Acs Photonics 2014, 1, 454–463. 10.1021/ph500032d.

[ref17] Tang C.; Auguié B.; Le Ru E. C. Refined effective-medium model for the optical properties of nanoparticles coated with anisotropic molecules. Phys. Rev. B 2021, 103, 085436 10.1103/PhysRevB.103.085436.

[ref18] Halas N. J.; Lal S.; Chang W.-S.; Link S.; Nordlander P. Plasmons in strongly coupled metallic nanostructures. Chem. Rev. 2011, 111, 3913–3961. 10.1021/cr200061k. 21542636

[ref19] Huang J.; Liu C.; Zhu Y.; Masala S.; Alarousu E.; Han Y.; Fratalocchi A. Harnessing structural darkness in the visible and infrared wavelengths for a new source of light. Nat. Nanotechnol. 2016, 11, 60–66. 10.1038/nnano.2015.228. 26479025

[ref20] Ngo N. M.; Tran H.-V.; Lee T. R. Plasmonic Nanostars: Systematic Review of their Synthesis and Applications. ACS Applied Nano Materials 2022, 5, 14051–14091. 10.1021/acsanm.2c02533.

[ref21] Lin T.; Yang T.; Cai Y.; Li J.; Lu G.; Chen S.; Li Y.; Guo L.; Maier S. A.; Liu C.; et al. others Transformation-Optics-Designed Plasmonic Singularities for Efficient Photocatalytic Hydrogen Evolution at Metal/Semiconductor Interfaces. Nano Lett. 2023, 23, 5288–5296. 10.1021/acs.nanolett.3c01287. 37234018 PMC10273458

[ref22] Byers C. P.; Zhang H.; Swearer D. F.; Yorulmaz M.; Hoener B. S.; Huang D.; Hoggard A.; Chang W.-S.; Mulvaney P.; Ringe E.; et al. From tunable core-shell nanoparticles to plasmonic drawbridges: Active control of nanoparticle optical properties. Sci. Adv. 2015, 1, e1500988 10.1126/sciadv.1500988. 26665175 PMC4672758

[ref23] Hammer B.; Nørskov J. K. Theoretical surface science and catalysis–calculations and concepts. Adv. Catal. 2000, 45, 71–129. 10.1016/S0360-0564(02)45013-4.

[ref24] Yamauchi M.; Kobayashi H.; Kitagawa H. Hydrogen storage mediated by Pd and Pt nanoparticles. ChemPhysChem 2009, 10, 2566–2576. 10.1002/cphc.200900289. 19823997

[ref25] Saldan I.; Semenyuk Y.; Marchuk I.; Reshetnyak O. Chemical synthesis and application of palladium nanoparticles. J. Mater. Sci. 2015, 50, 2337–2354. 10.1007/s10853-014-8802-2.

[ref26] Pedone D.; Moglianetti M.; De Luca E.; Bardi G.; Pompa P. P. Platinum nanoparticles in nanobiomedicine. Chem. Soc. Rev. 2017, 46, 4951–4975. 10.1039/C7CS00152E. 28696452

[ref27] Guo J.; Zhang Y.; Shi L.; Zhu Y.; Mideksa M. F.; Hou K.; Zhao W.; Wang D.; Zhao M.; Zhang X.; et al. Boosting hot electrons in hetero-superstructures for plasmon-enhanced catalysis. J. Am. Chem. Soc. 2017, 139, 17964–17972. 10.1021/jacs.7b08903. 29155572

[ref28] Aslam U.; Chavez S.; Linic S. Controlling energy flow in multimetallic nanostructures for plasmonic catalysis. Nat. Nanotechnol. 2017, 12, 1000–1005. 10.1038/nnano.2017.131. 28737751

[ref29] Vadai M.; Angell D. K.; Hayee F.; Sytwu K.; Dionne J. A. In-situ observation of plasmon-controlled photocatalytic dehydrogenation of individual palladium nanoparticles. Nat. Commun. 2018, 9, 4658 10.1038/s41467-018-07108-x. 30405133 PMC6220256

[ref30] Sytwu K.; Vadai M.; Dionne J. A. Bimetallic nanostructures: combining plasmonic and catalytic metals for photocatalysis. Advances in Physics: X 2019, 4, 1619480 10.1080/23746149.2019.1619480.

[ref31] Zhou L.; Martirez J. M. P.; Finzel J.; Zhang C.; Swearer D. F.; Tian S.; Robatjazi H.; Lou M.; Dong L.; Henderson L.; et al. Light-driven methane dry reforming with single atomic site antenna-reactor plasmonic photocatalysts. Nature Energy 2020, 5, 61–70. 10.1038/s41560-019-0517-9.

[ref32] Sytwu K.; Vadai M.; Hayee F.; Angell D. K.; Dai A.; Dixon J.; Dionne J. A. Driving energetically unfavorable dehydrogenation dynamics with plasmonics. Science 2021, 371, 280–283. 10.1126/science.abd2847. 33446555

[ref33] Ezendam S.; Herran M.; Nan L.; Gruber C.; Kang Y.; Groebmeyer F.; Lin R.; Gargiulo J.; Sousa-Castillo A.; Cortés E. Hybrid plasmonic nanomaterials for hydrogen generation and carbon dioxide reduction. ACS Energy Letters 2022, 7, 778–815. 10.1021/acsenergylett.1c02241. 35178471 PMC8845048

[ref34] Rodrigues M. P. d. S.; Dourado A. H.; Cutolo L. d. O.; Parreira L. S.; Alves T. V.; Slater T. J.; Haigh S. J.; Camargo P. H.; Cordoba de Torresi S. I. Gold–rhodium nanoflowers for the plasmon-enhanced hydrogen evolution reaction under visible light. ACS Catal. 2021, 11, 13543–13555. 10.1021/acscatal.1c02938.

[ref35] Yuan L.; Zhou J.; Zhang M.; Wen X.; Martirez J. M. P.; Robatjazi H.; Zhou L.; Carter E. A.; Nordlander P.; Halas N. J. Plasmonic Photocatalysis with Chemically and Spatially Specific Antenna–Dual Reactor Complexes. ACS Nano 2022, 16, 17365–17375. 10.1021/acsnano.2c08191. 36201312

[ref36] Herran M.; Sousa-Castillo A.; Fan C.; Lee S.; Xie W.; Döblinger M.; Auguié B.; Cortés E. Tailoring plasmonic bimetallic nanocatalysts toward sunlight-driven H2 production. Adv. Funct. Mater. 2022, 32, 2203418 10.1002/adfm.202203418.

[ref37] Gargiulo J.; Herran M.; Violi I. L.; Sousa-Castillo A.; Martinez L. P.; Ezendam S.; Barella M.; Giesler H.; Grzeschik R.; Schlücker S.; et al. Impact of bimetallic interface design on heat generation in plasmonic Au/Pd nanostructures studied by single-particle thermometry. Nat. Commun. 2023, 14, 3813 10.1038/s41467-023-38982-9. 37369657 PMC10300195

[ref38] Herran M.; Juergensen S.; Kessens M.; Hoeing D.; Köppen A.; Sousa-Castillo A.; Parak W. J.; Lange H.; Reich S.; Schulz F.; et al. Plasmonic bimetallic two-dimensional supercrystals for H2 generation. Nat. Catal. 2023, 6, 1205 10.1038/s41929-023-01053-9.

[ref39] Benisty H.; Greffet J.-J.; Lalanne P. Introduction to Nanophotonics; Oxford University Press, 2022.

[ref40] Bohren C. F.; Huffman D. R. Asorption and Scattering of Light by Small Particles; John Wiley & Sons, 2008.

[ref41] Chettiar U. K.; Engheta N. Internal homogenization: Effective permittivity of a coated sphere. Opt. Express 2012, 20, 22976–22986. 10.1364/OE.20.022976. 23188261

[ref42] Neeves A. E.; Birnboim M. H. Composite structures for the enhancement of nonlinear-optical susceptibility. JOSA B 1989, 6, 787–796. 10.1364/JOSAB.6.000787. 19746133

[ref43] Choy T. C. Effective Medium Theory: Principles and Application International Series of Monographs on Physics, series vol. 165; Oxford University Press, 2015.

[ref44] Maxwell J. C.; Garnett B. XII. Colours in metal glasses and in metallic films. Philos. Trans. R. Soc., A 1904, 203, 385–420. 10.1098/rsta.1904.0024.

[ref45] Markel V. A. Introduction to the Maxwell Garnett approximation: tutorial. JOSA A 2016, 33, 1244–1256. 10.1364/JOSAA.33.001244. 27409680

[ref46] Liu C.; Di Falco A.; Molinari D.; Khan Y.; Ooi B. S.; Krauss T. F.; Fratalocchi A. Enhanced energy storage in chaotic optical resonators. Nat. Photonics 2013, 7, 473–478. 10.1038/nphoton.2013.108.

[ref47] Lalisse A.; Tessier G.; Plain J.; Baffou G. Quantifying the efficiency of plasmonic materials for near-field enhancement and photothermal conversion. J. Phys. Chem. C 2015, 119, 25518–25528. 10.1021/acs.jpcc.5b09294.

[ref48] Pendry J.; Huidobro P. A.; Luo Y.; Galiffi E. Compacted dimensions and singular plasmonic surfaces. Science 2017, 358, 915–917. 10.1126/science.aap7939. 29146809

[ref49] Zhang J.; Pendry J. B.; Luo Y. Transformation optics from macroscopic to nanoscale regimes: a review. Advanced Photonics 2019, 1, 014001 10.1117/1.AP.1.1.014001.

[ref50] Wu T.; Li K.; Zhang N.; Xia J.; Zeng Q.; Wen X.; Dinish U. S.; Olivo M.; Shen Z.; Liu Z.; et al. Ultrawideband surface enhanced Raman scattering in hybrid graphene fragmented-gold substrates via cold-etching. Adv. Opt. Mater. 2019, 7, 1900905 10.1002/adom.201900905.

[ref51] Hu Z.; Liu C.; Li G. Disordered optical metasurfaces: from light manipulation to energy harvesting. Adv. Phys.: X 2023, 8, 2234136 10.1080/23746149.2023.2234136.

[ref52] Lalanne P.; Dmitriev A.; Rockstuhl C.; Sprafke A.; Vynck K. Minireview on Disordered Optical Metasurfaces. arXiv (physics.optics), 22 Oct 2023, arXiv:2310.14349, ver. 2. DOI: 10.48550/arXiv.2310.14349 (accessed 2024–03–26).

[ref53] Chikkaraddy R.; De Nijs B.; Benz F.; Barrow S. J.; Scherman O. A.; Rosta E.; Demetriadou A.; Fox P.; Hess O.; Baumberg J. J. Single-molecule strong coupling at room temperature in plasmonic nanocavities. Nature 2016, 535, 127–130. 10.1038/nature17974. 27296227 PMC4947385

[ref54] Liu L.; Tobing L. Y.; Wu T.; Qiang B.; Garcia-Vidal F. J.; Zhang D. H.; Wang Q. J.; Luo Y. Plasmon-induced thermal tuning of few-exciton strong coupling in 2D atomic crystals. Optica 2021, 8, 1416–1423. 10.1364/OPTICA.436140.

[ref55] Hartland G. V.; Besteiro L. V.; Johns P.; Govorov A. O. What’s so hot about electrons in metal nanoparticles?. ACS Energy Letters 2017, 2, 1641–1653. 10.1021/acsenergylett.7b00333.

[ref56] Lee H.; Lim J.; Lee C.; Back S.; An K.; Shin J. W.; Ryoo R.; Jung Y.; Park J. Y. Boosting hot electron flux and catalytic activity at metal–oxide interfaces of PtCo bimetallic nanoparticles. Nat. Commun. 2018, 9, 2235 10.1038/s41467-018-04713-8. 29884825 PMC5993833

[ref57] Martinez L. P.; Poklepovich-Caride S.; Gargiulo J.; Martínez E. D.; Stefani F. D.; Angelomé P. C.; Violi I. L. Optical Printing of Single Au Nanostars. Nano Lett. 2023, 23, 2703–2709. 10.1021/acs.nanolett.2c05109. 36952678

[ref58] Pallares R. M.; Stilson T.; Choo P.; Hu J.; Odom T. W. Using good’s buffers to control the anisotropic structure and optical properties of spiky gold nanoparticles for refractive index sensing. ACS Appl. Nano Mater. 2019, 2, 5266–5271. 10.1021/acsanm.9b01117.

[ref59] Jia J.; Metzkow N.; Park S.-M.; Wu Y. L.; Sample A. D.; Diloknawarit B.; Jung I.; Odom T. W. Spike Growth on Patterned Gold Nanoparticle Scaffolds. Nano Lett. 2023, 23, 11260–11265. 10.1021/acs.nanolett.3c03778. 38048438

[ref60] Yi C.; Liu H.; Zhang S.; Yang Y.; Zhang Y.; Lu Z.; Kumacheva E.; Nie Z. Self-limiting directional nanoparticle bonding governed by reaction stoichiometry. Science 2020, 369, 1369–1374. 10.1126/science.aba8653. 32913102

[ref61] Sokołowski K.; Huang J.; Földes T.; McCune J. A.; Xu D. D.; de Nijs B.; Chikkaraddy R.; Collins S. M.; Rosta E.; Baumberg J. J.; et al. others Nanoparticle surfactants for kinetically arrested photoactive assemblies to track light-induced electron transfer. Nat. Nanotechnol. 2021, 16, 1121–1129. 10.1038/s41565-021-00949-6. 34475556

[ref62] Li Z.; Young N.; Di Vece M.; Palomba S.; Palmer R.; Bleloch A.; Curley B.; Johnston R.; Jiang J.; Yuan J. Three-dimensional atomic-scale structure of size-selected gold nanoclusters. Nature 2008, 451, 46–48. 10.1038/nature06470. 18066049

[ref63] Mao P.; Liu C.; Niu Y.; Qin Y.; Song F.; Han M.; Palmer R. E.; Maier S. A.; Zhang S. Disorder-Induced Material-Insensitive Optical Response in Plasmonic Nanostructures: Vibrant Structural Colors from Noble Metals. Adv. Mater. 2021, 33, 2007623 10.1002/adma.202007623. 33929067

[ref64] Mao P.; Liu C.; Chen Q.; Han M.; Maier S. A.; Zhang S. Broadband SERS detection with disordered plasmonic hybrid aggregates. Nanoscale 2020, 12, 93–102. 10.1039/C9NR08118F. 31674618

[ref65] Li X.; Chen Q.; McCue I.; Snyder J.; Crozier P.; Erlebacher J.; Sieradzki K. Dealloying of noble-metal alloy nanoparticles. Nano Lett. 2014, 14, 2569–2577. 10.1021/nl500377g. 24689459

[ref66] Kim M.; Ko S. M.; Nam J.-M. Dealloying-based facile synthesis and highly catalytic properties of Au core/porous shell nanoparticles. Nanoscale 2016, 8, 11707–11717. 10.1039/C6NR01321J. 27221241

[ref67] Zhang N.; Han C.; Xu Y.-J.; Foley J. J. IV; Zhang D.; Codrington J.; Gray S. K.; Sun Y. Near-field dielectric scattering promotes optical absorption by platinum nanoparticles. Nat. Photonics 2016, 10, 473–482. 10.1038/nphoton.2016.76.

[ref68] Mao P.; Liu C.; Favraud G.; Chen Q.; Han M.; Fratalocchi A.; Zhang S. Broadband single molecule SERS detection designed by warped optical spaces. Nat. Commun. 2018, 9, 5428 10.1038/s41467-018-07869-5. 30575738 PMC6303368

[ref69] Ciracì C.; Hill R.; Mock J.; Urzhumov Y.; Fernández-Domínguez A.; Maier S.; Pendry J.; Chilkoti A.; Smith D. Probing the ultimate limits of plasmonic enhancement. Science 2012, 337, 1072–1074. 10.1126/science.1224823. 22936772 PMC3649871

[ref70] Yang Y.; Zhu D.; Yan W.; Agarwal A.; Zheng M.; Joannopoulos J. D.; Lalanne P.; Christensen T.; Berggren K. K.; Soljačić M. A general theoretical and experimental framework for nanoscale electromagnetism. Nature 2019, 576, 248–252. 10.1038/s41586-019-1803-1. 31827292

