# Supplementary material for: Enhanced Light–Matter Interaction in Metallic Nanoparticles: A Generic Strategy of Smart Void Filling
Source: Nano Lett. 2024 Apr 5;24(15):4641–8. doi: 10.1021/acs.nanolett.4c00810 (PMC11036389; doi:10.1021/acs.nanolett.4c00810)
Supplement: Supplementary file 1 — nl4c00810_si_001.pdf [file nl4c00810_si_001.pdf]

Supplementary Materials for "Enhanced light matter interaction in metallic nanoparticles - a generic strategy of smart void filling"

Changxu Liu,[∗,†] Tong Wu,[‡] Philippe Lalanne,[‡] and Stefan A. Maier[∗,¶,§]

†*Centre for Metamaterial Research & Innovation, Department of Engineering, University of Exeter, Exeter EX4 4QF, United Kingdom*

‡*LP2N, Institut d'Optique Graduate School, CNRS, Université de Bordeaux, Talence, 33400, France*

¶*School of Physics and Astronomy, Monash University, Clayton, Victoria 3800, Australia*

§*Blackett Laboratory, Imperial College London, London SW7 2BZ, United Kingdom*

E-mail: c.c.liu@exeter.ac.uk; stefan.maier@monash.edu

1. The trajectory of void filling under different wavelengths for Au nanosphere

Here, we demonstrate the trajectories of n_{eff} in the complex refractive index plane at different wavelengths. The results are demonstrated in Fig.S1. The value of n mainly determines the value of D defined in the main text. When the value of $n > 1$ (S1a,b), the trajectories is not able to approaching regions with large values of Q_{ext}.

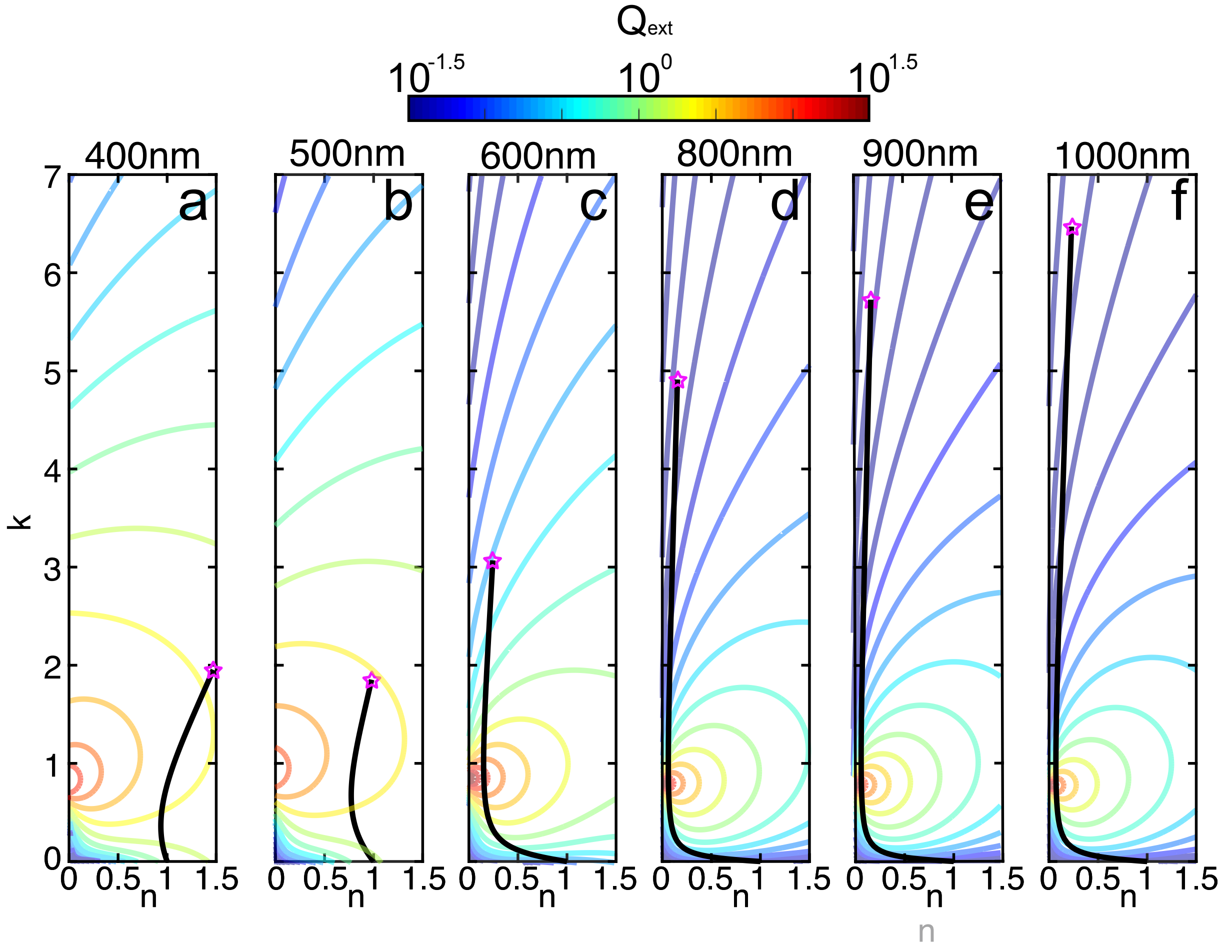

Figure S1: The trajectory of effective refractive index (n_{eff}) from void filling at different wavelengths. (a) 400 nm; (b) 500 nm; (c) 600 nm; (d) 800 nm; (e) 900 nm; (f) 1000 nm. The background is a contour representing the extinction efficiency Q_{ext}.

2. The universal adaptability of smarting void filling - calculation based on Mie theory

In the manuscript, we demonstrate the effect of void filling using a core-shell structure with a thin shell, or equivalently, a small value of P. In Fig. S2, we show two additional configurations with different core sizes (R_i=15nm and 30nm) while keeping the overall particle size constant (R_o=65nm). Notable enhancements in extinction are observed with different values of P, as shown in Fig. S2 a and c. Correspondingly, Fig. S2 b and d show the trajectory of n_{eff} when prominent enhancement occurs at λ=700. A shift of the resonant point $\tilde{n}_r$ in the plane is observed, compared to Fig.1b in the manuscript. However, the void filling retains its smart feature at various P - securing the trajectory of the effective index going through the region with enhanced light-matter interaction. From Eq.(1) and (2) in the manuscript, the extinction efficiency is independent of the particle size. Therefore, the enhancement can be achieved at varoius values of R_o and P.

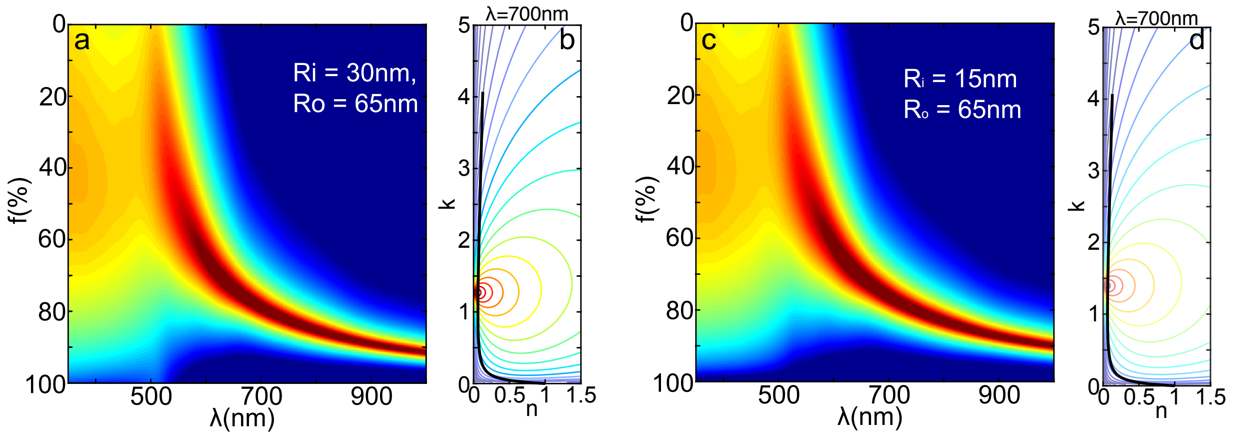

Figure S2: The smart voiding filling with different core particle sizes. (a,b) R_i=30nm and R_o=65nm; (c,d) R_i=15nm and R_o=65nm. (a) and (b) illustrate the extinction efficiency (Q_{ext}) spectra at different filling factors (f). (c) and (d) illustrate the trajectories of n_{eff} at λ=700nm. The background is a contour represent the extinction efficiency Q_{ext}. The colour map and contour are the same as the ones shown in Fig. 1 and Fig. 2 in the manuscript.

We further investigate the impact of the host material. For the simplicity but not losing generality, we replace the value of the background refractive index from 1 to 1.33, with

results shown in Fig. S3. Similarly, Fig. S3 shows a prominent enhancement, with a red shift of the region under large enhancement of Q_{ext}. Again, the effect of the void filling is maintained, as demonstrated in Fig. S3. The ending point of the trajectory is shifted to 1.33, as we assume the voids have the same refractive index of the background.

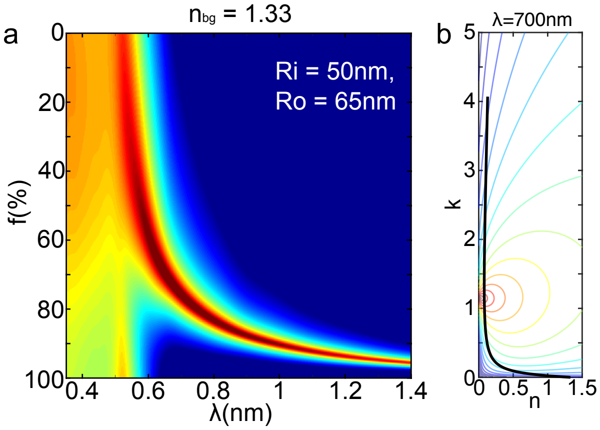

Figure S3: The smart voiding filling in a different host material with n_{bg}=1.33. (a) Extinction efficiency (Q_{ext}) spectra at different filling factors (f). (b) The trajectory of n_{eff} at λ=700 nm. The background in (c) is a contour represent the extinction efficiency Q_{ext}. The colour map and contour are the same as the ones shown in Fig. 1 and Fig. 2 in the manuscript.

3. Refractive indices for the transition metals

We illustrate the refractive indices of the eight metal used in the manuscript. The black dashed lines are an eye guide of the value of 0.5. Notable enhancement of Q_{ext} is observed when the value of k is small, as shown in Fig.3 in the manuscript. The value of n and k are obtained from ref.[1] for Au and ref[2] for rest of the metals.

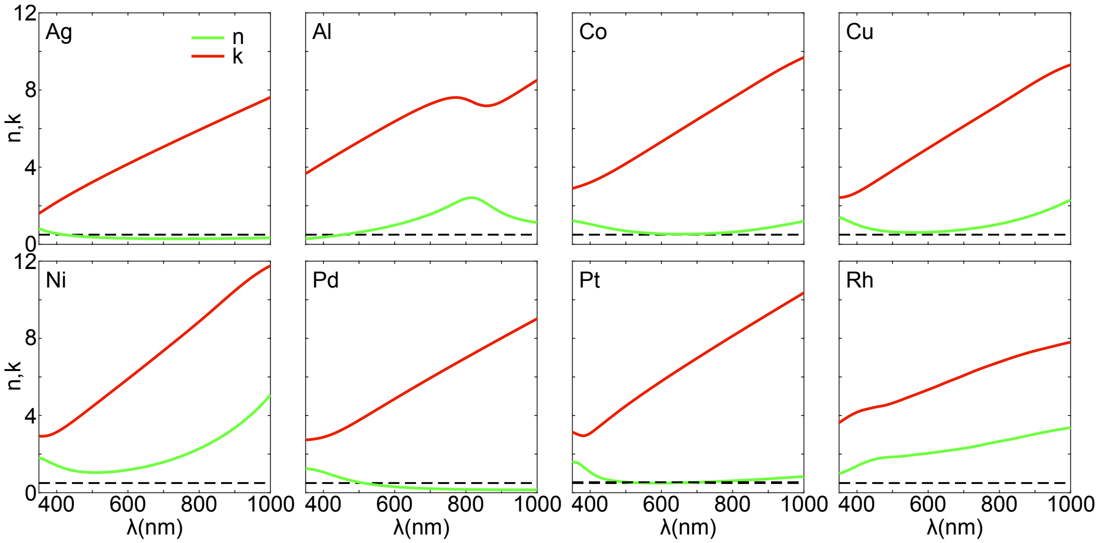

Figure S4: The complex refractive indecies of various transition metals. (a) Ag; (b) Al; (c) Co; (d) Cu; (e) Ni; (f) Pd; (g) Pt; (h) Rh.

4. The extinction efficiency and scattering efficiency for nano-urchins composed of different metals

Based on FDTD simulations, the extinction efficiency and scattering efficiency are demonstrated in Fig. S5 and Fig. S6, respectively.

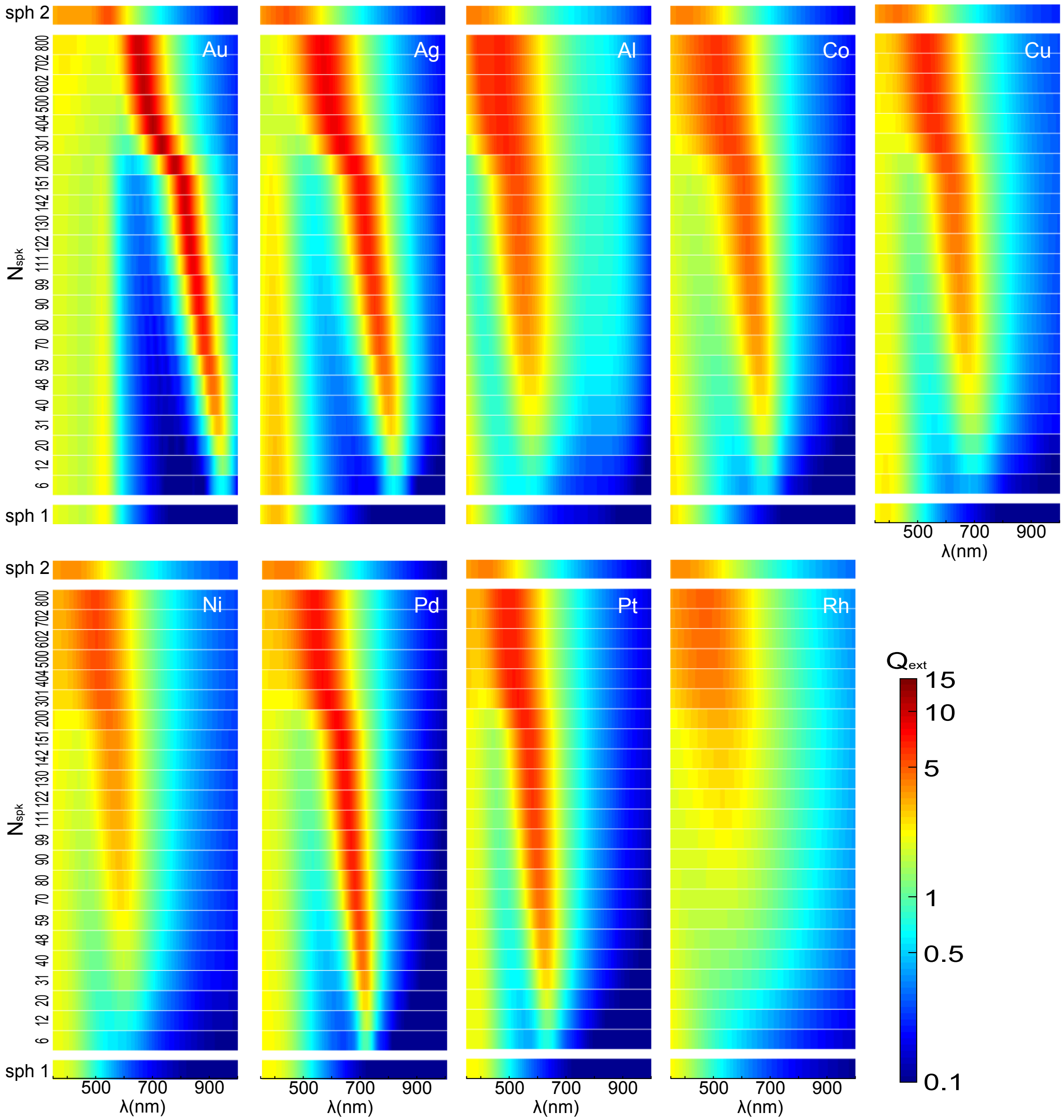

Figure S5: The spectra of extinction efficiency Q_{ext} at different f for nine metals. The type of metal is list on the top right in each panel.

We observe a similar phenomenon of the enhancement in scattering efficiency. The en-

hancement is achieved for all nine metals, with the possibility to shift scattering peaks to larger wavelengths. Therefore, the voiding filling can boost both absorption and scattering.

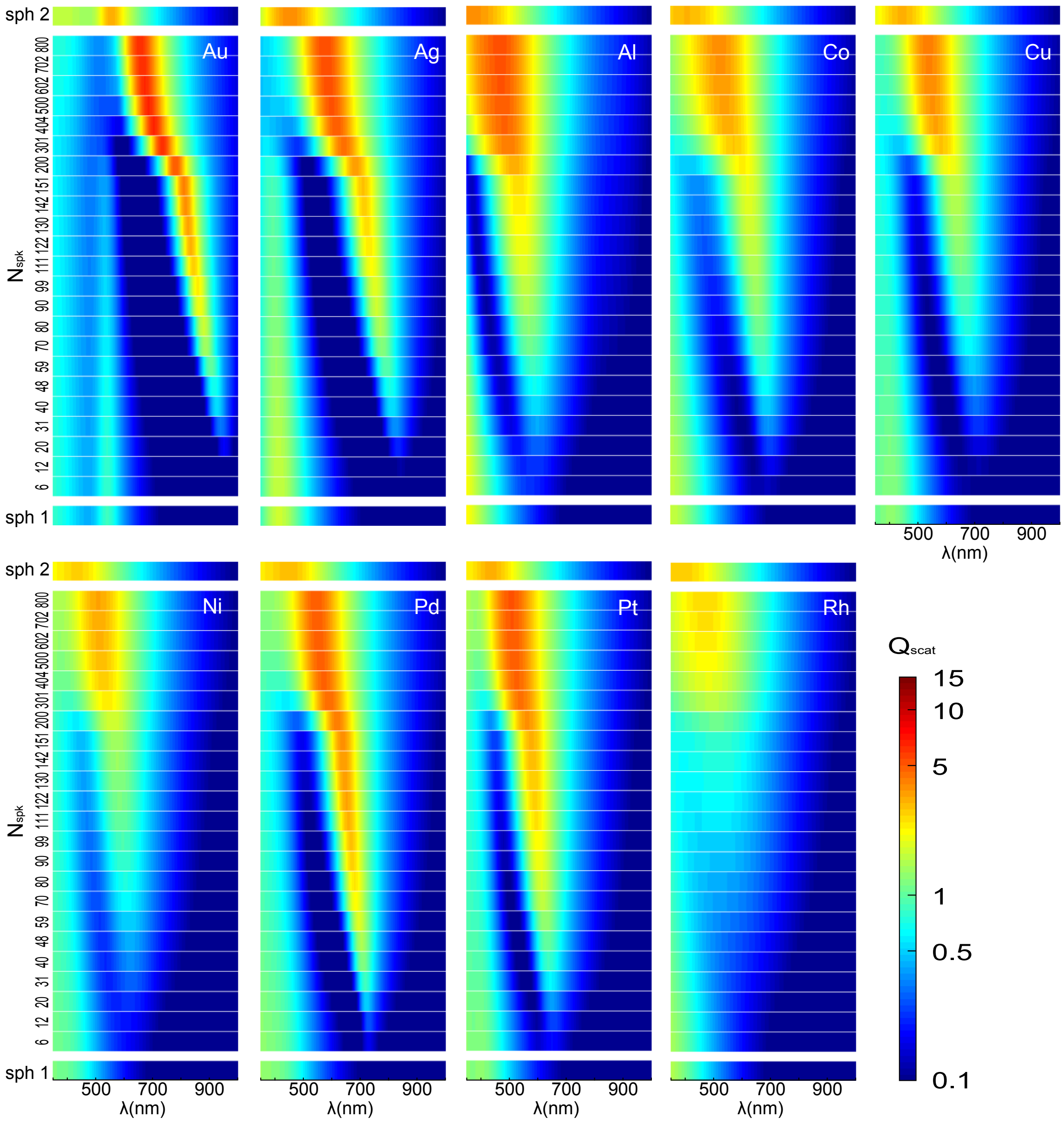

Figure S6: The spectra of scattering efficiency Q_{scat} at different f for nine metals. The type of metal is list on the top right in each panel.

5. Details of the simulations

General information of the FDTD simulations. The FDTD method is implemented using the commercialised software Lumerical. The refractive indices of the nine metals share the same values in calculations based on Mie Theory.

The calculation of cross-sections is performed through the analysis group with a Total-field Scattered-field (TFSF) source provided by Lumerical. Perfectly matched layer (PML) is used for boundary layers. The mesh size is selected as 1nm in all x,y,z directions.

Both the structures of nano-urchin and nano-berry are imported from stl files. All stl files are developed from Comsol Multiphysics, where a LiveLink for MATLAB is used. Consequently, the geometry can be controlled and produced through MATLAB scripts. The algorithm to produce uniform distributed points on a sphere is from ref.[3]

Simulations of nano-urchins. The geometry of an individual spike is demonstrated in Fig. S7 The total height of the spike is 5 nm greater than the thickness of the shell ($R_o - R_i$), accounting for a 5 nm overlap between the spike and the core. The spike are uniformly distributed on the surface of the core sphere. And the orientation of each spike is along the same direction of the radius of the core sphere.

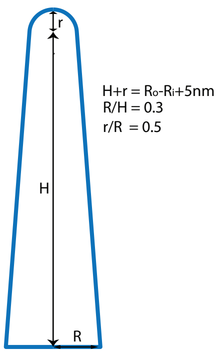

Figure S7: The geometry of the spike used in nano-urchins.

Simulations of disordered nano-urchins. We introduce geometric disorder to the nano-urchin with the maximum Q_{abs}. Two types of disorder are included: the deformation of shape and the fluctuation of the orientation of the spikes.

A parameter, δ, is introduced to represent the level of the disorder. The original spike, as shown in Fig.S7, undergoes a re-scaling in all x, y, and z directions, with scale factors $[1+\delta U_x, 1+\delta U_y, 1+\delta U_z]$. The directions of x, y and z are demonstrated in Fig.S7, respectively. The terms $1+\delta U_x$ and $1+\delta U_y$ re-scale the shape of the spike within the transverse x-y plane, while the term $1+\delta U_z$ modifies the height of the spike. Additionally, the orientation of spike changes from its initial direction $[\theta_0, \phi_0]$ to $[\theta_0 + \frac{\pi}{2}\delta U_\theta, \phi_0 + \frac{\pi}{2}\delta U_\phi]$, where θ_0 is the altitude angle and ϕ_0 is the azimuth angle. Here, U_x, U_y, U_z, U_θ and U_ϕ are independent random variables with uniform distribution in the range [-1, 1], generated by rand() function from MATLAB.

Simulations of nano-blackberries. The core is a 100 nm sphere. The size of the outer spheres are chosen as 17 nm, uniformly allocated on the surface of the core. There is 2 nm overlap between the core and the outer spheres, to model the realistic situations (no point contacts).

6. Impact of disorder on the effect of void filling - another set of random variables

Here we provide simulations using a different set of random variables, as shown in Fig. S8. Again, the parameter δ presents the level of disorder. Similarly, the broadening of the absorption is observed. Despite the difference in the details of the spectra, the disorder does not reduce the enhancement of averaged value within the spectrum between 350 nm and 1000 nm. The variation of averaged Q_{abs} between 350 to 1000nm is 103.6% for $\delta = 0.1$, 108.9% for $\delta = 0.2$ and 101.1% for $\delta = 0.4$, respectively.

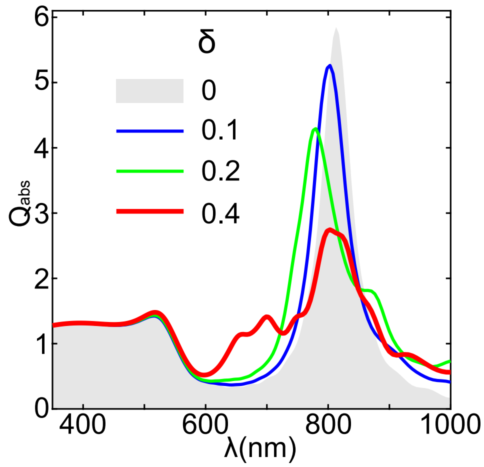

Figure S8: The spectra of Q_{abs} under the existence of the disorder. Another set of random variables are used compared to Fig.4c in the manuscript.

7. Impact of polarisation of incident light

Here, we investigate the polarisation dependence of the nano-urchin. The electric field lies within the x-y plane, rotated by an angle of θ from x-axis, while the wavevector is along z-axis.

We choose two sets with different levels of disorder and the results are summarised in Fig. S9. The increment of the disorder can reduce the symmetry of the structure, leading to an increase in the Q_{abs} difference among various polarisations as δ goes from 0.1 (Fig. S9a) to 0.4 (Fig. S9b). However, even under high levels of disorder (Fig. S9b), the nano-urchin structure exhibits a good polarization insensitivity, as shown in Fig. S9b.

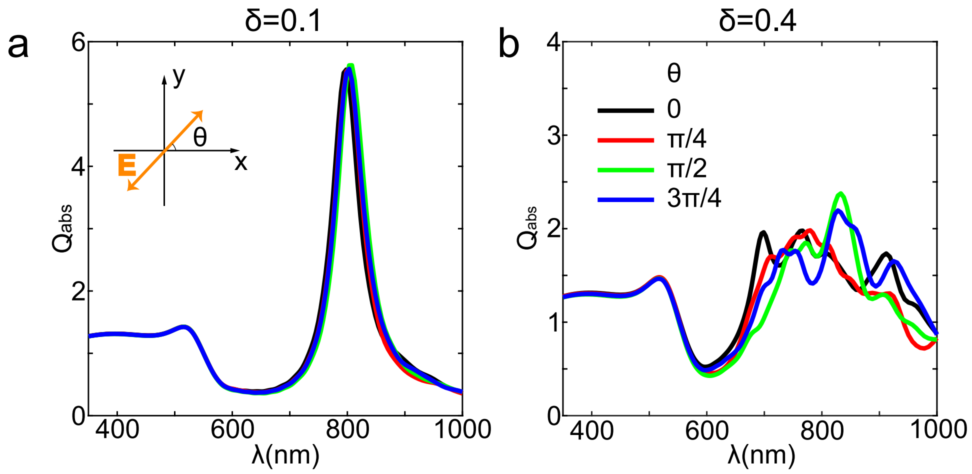

Figure S9: The spectra of Q_{abs} under different polarisations of the incident light. Two sets are selected with different values of δ, (a) $\delta = 0.1$ and (b) $\delta = 0.4$. The inset in (a) illustrates the direction of the electric field $\boldsymbol{E}$, which lies within the x-y plane at an angle of *theta* to the x-axis. The directions of the x, y and z axes are shown in Fig. 4c of the manuscript.

8. Impact of dielectric coating

Here, we explore a practical case for metallic nanoparticles coated with dielectric layers. A dielectric shell may play a role as a passivisation layer for protection. More importantly, wide-band semiconductor shell can operate as a functional layer for specific applications, such as the catalyst for photocatalysis. We have selected three distinct dielectric materials: SiO_2, WO_3, TiO_2. While silica is commonly used for protection, WO_3 and TiO_2 show promise in photocatalysis.[4,5] The refractive indices for these materials are chosen as 1.45 (SiO_2),[6] 1.97(WO_3)[7] and 2.55 (TiO_2),[8] respectively.

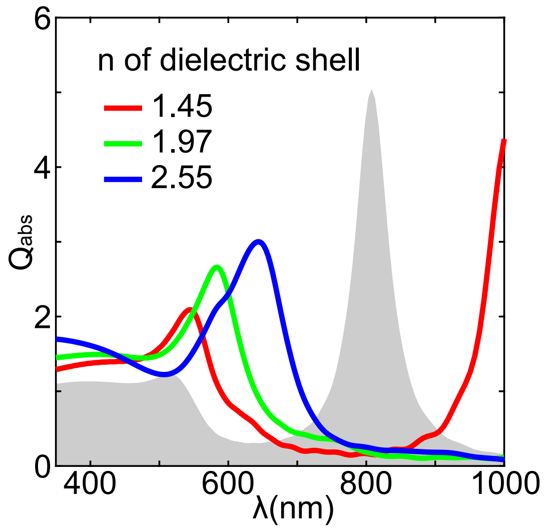

Figure S10: The spectra of Q_{abs} with a dielectric coating. Spherical shells with different refractive index n are investigated. The radius of the shell is 70nm. The grey shaded area is the one without coating.

Again, we select the particle as the Au nanourchin with $N_{\mathrm{spk}} = 151$. We assume the dielectric shell is spherical and the radius is 70nm. The Q_{abs} spectra are calculated through FDTD simulations, as shown in Fig. S10. The introduction of the dielectric coating alters the optical environment, causing shifts in the nanoparticles' resonances. Interestingly, the coating improve the absorption in the visible region, which is advantageous for applications like solar hydrogen generation and photovoltaics. The averaged values of Q_{abs} between 350nm to 1000nm does not experiences prominent variations, with a 82.4% (n=1.45),

99.9%(n=1.97) and 112.1%(n=2.55) change compared to the same particle without shell, respectively. Beyond its own chemical and/or electrical properties, the choice of dielectric material for coating offers the opportunity to customise the nanoparticle's response to match specific light sources.

9. Nanoblackberry - alternative design for a realisable naoprticle with void filling

Here we provide a secondary realisable design, as shown in Fig. S11. Secondary nanospheres are affixed to the surface of the core, forming a structures resembling blackberries. The spectra of Q_{abs} are simulated with varying numbers of secondary spheres N_{sph}, with results presented in Fig.S11b. While similar level of absorption enhancement is maintained, the red shift of the peak is not that pronounced, especially for cases with $N_{\mathrm{sph}} > 31$. We attribute the difference between the Mie model and simulations to the non-uniformity of the void filling and additional resonant features of the spheres or spikes. More details of the FDTD simulations for nano-urchins and nano-blackberries can be found in Supplementary Section 8.

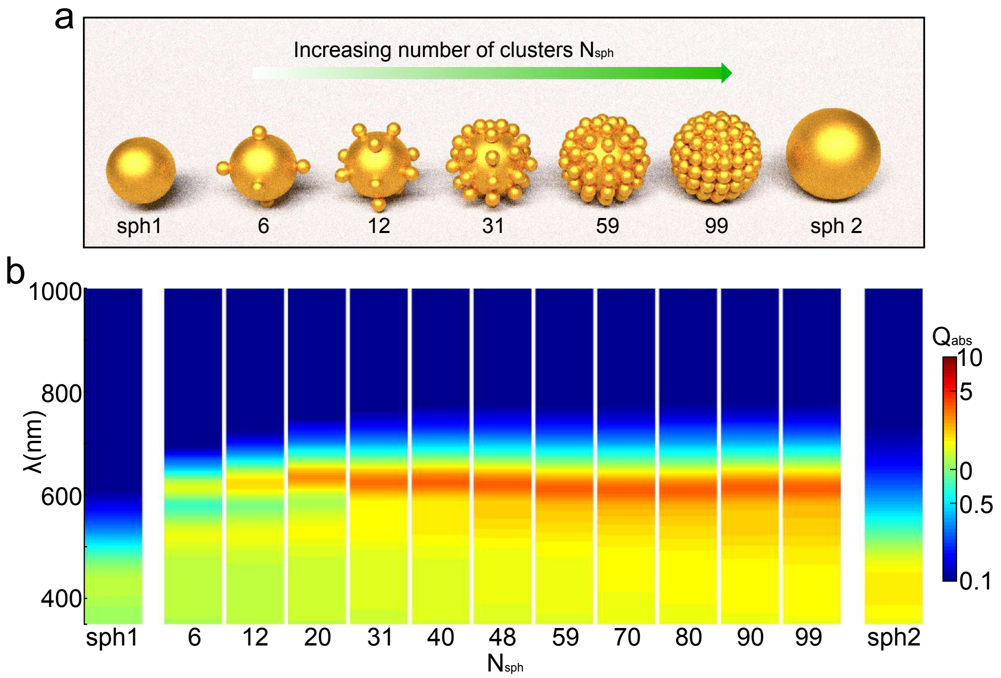

Figure S11: An alternative design for the realisation of void filling. (a) The schematic of nano-blackberries. The filling factor (f) can be tuned by varying the numbers of secondary nanospheres (N_{sph}). (b) The Q_{abs} spectra for nano-blackberries with different N_{sph}.

10. A comparison between nano-blackberry and nano-urchin

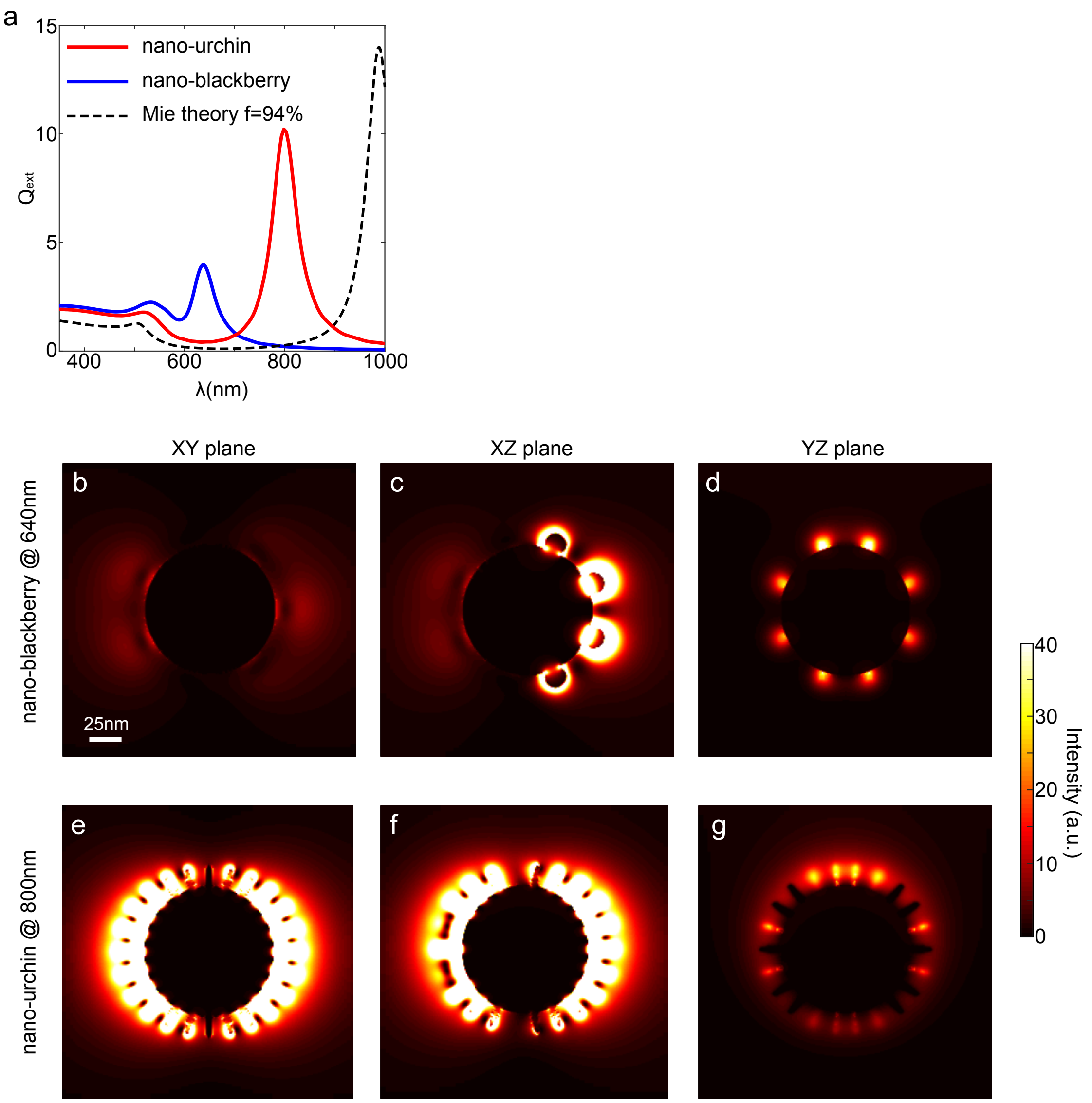

Figure S12: A comparison between nano-urchin and nano-blackberry. The nano-urchin consists of 168 spikes, whereas the nano-blackberry comprises 20 secondary spheres. Both nanoparticles exhibit a filling factor of $f = 94\%$ of air in the shell. (a) The spectra of $Q_{\rm abs}$ for nano-urchin, nano-blackberry and the one from Mie Theory. (b-d) Spatial distribution of light intensity $|E|^2$ for nano-blackberry at 640nm. The cross-sections along the (b)XY plane, (c) XZ plane and (d) YZ plane are shown respectively. (e-g) Spatial distribution of light intensity $|E|^2$ for nano-urchin at 800nm. The cross-sections along the (e)XY plane, (f) XZ plane and (g) YZ plane are shown respectively. All cross-sections pass through the centre of the core sphere. The indecent light light is directed along the negative z direction.

In this section, we conduct a quantitative comparison between nano-blackberry and nano-urchin, selecting configurations to achieve the same filling factor of 94%. The Q_{abs} spectra are demonstrated in Fig. S12a. The spectrum from Mie theory is also included, plotted as the black dashed line. Owing to the nonuniform void filling, we observe resonance shifts and peak degradation, particularly pronounced in nano-blackberry due to stronger non-uniformity.

We also investigate the electromagnetic response in the near-field, with results summarised in Fig. S12b-g. The spatial distributions of intensity on three orthogonal cross-sections are plotted respectively at the resonance wavelength, with light propagation along the negative z-axis The polarisation is along x-axis, inducing a stronger field enhancement in the XZ plane (Fig. S12c and f) than in the YZ plane (Fig. S12d and g). The specific features, such as spikes for nano-urchin and secondary nanospheres for nano-blackberry, significantly influence the optical response. The absorption peaks coincides the plasmonic resonances of the spikes and spheres, demonstrated by strong nearfield enhancement. Conversely, within cross-sections lacking spikes or nanospheres, intensity enhancement is limited.

Consequently, the specific morphology of nanoparticles significantly contributes to their optical response. Even nanoparticles with the same filling factor may exhibit distinct absorption/scattering spectra, deviating from predictions of the Mie model, which assumes uniform distribution of voids orders smaller than the wavelength in free space.

11. A simple estimation for the reduction of material usage in absorption-related applications

Here, we calculate the amount of material needed for achieving a desired absorption of 90%, using Beer–Lambert law. We assume uniform distribution of particles in a host material ($n_{\text{bg=1}}$) with a thickness of 1 cm and area of 1m^2. The mass of the metals (M_{90}) are shown in Fig.S13, covering the visible and near-infrared spectrum. We choose the nanourchin with maximum Q_{abs} for each metal respectively (Fig. 3b in the manuscript). Due to the enhanced light-matter interacting, one order of magnitude less material is needed at optimised wavelengths.

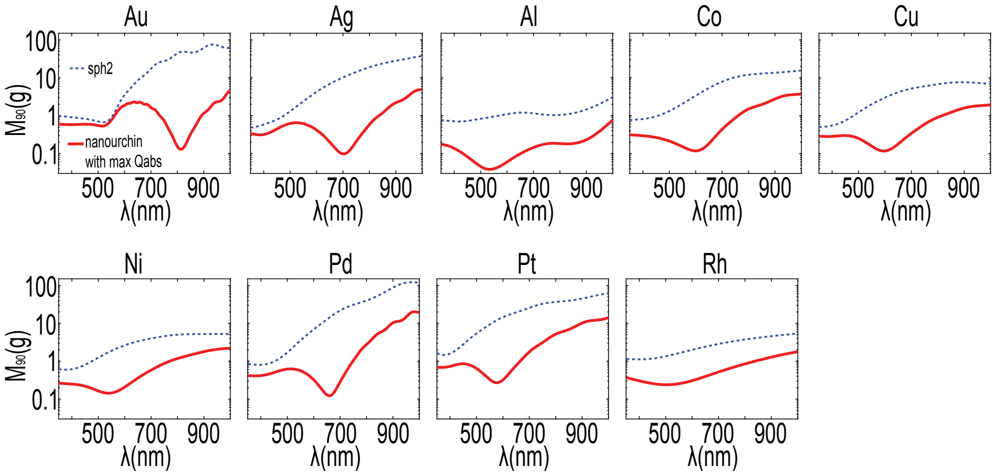

Figure S13: The material consumption (M_{90}) to achieve 90% absorption for different nanourchins. The blue dashed line serves as a reference for spherical nanoparicles with the same size. The type of the metal is label at the top in each panel.

Here, we make a conservative estimation, as the scattering and consequently the secondary absorption are not accounted. Considering the enhanced scattering from void filling, the material usages may be further reduced, as the scattered photons can be further captured by secondary nanoparticles.

References

(1) Johnson, P. B.; Christy, R.-W. Optical constants of the noble metals. *Physical review B* **1972**, *6*, 4370.

(2) Werner, W. S.; Glantschnig, K.; Ambrosch-Draxl, C. Optical constants and inelastic electron-scattering data for 17 elemental metals. *Journal of Physical and Chemical Reference Data* **2009**, *38*, 1013–1092.

(3) Deserno, M. How to generate equidistributed points on the surface of a sphere. *If Polymerforshung (Ed.)* **2004**, *99*, 1.

(4) Xiang, Q.; Meng, G.; Zhao, H.; Zhang, Y.; Li, H.; Ma, W.; Xu, J. Au nanoparticle modified WO3 nanorods with their enhanced properties for photocatalysis and gas sensing. *The Journal of Physical Chemistry C* **2010**, *114*, 2049–2055.

(5) Murdoch, M.; Waterhouse, G.; Nadeem, M.; Metson, J.; Keane, M.; Howe, R.; Llorca, J.; Idriss, H. The effect of gold loading and particle size on photocatalytic hydrogen production from ethanol over Au/TiO2 nanoparticles. *Nature chemistry* **2011**, *3*, 489–492.

(6) Malitson, I. H. Interspecimen comparison of the refractive index of fused silica. *Josa* **1965**, *55*, 1205–1209.

(7) Kulikova, D. P.; Dobronosova, A. A.; Kornienko, V. V.; Nechepurenko, I. A.; Baburin, A. S.; Sergeev, E. V.; Lotkov, E. S.; Rodionov, I. A.; Baryshev, A. V.; Dorofeenko, A. V. Optical properties of tungsten trioxide, palladium, and platinum thin films for functional nanostructures engineering. *Optics express* **2020**, *28*, 32049–32060.

(8) DeVore, J. R. Refractive indices of rutile and sphalerite. *JOSA* **1951**, *41*, 416–419.
